# Automatic Spiral Analysis for Objective Assessment of Motor Symptoms in Parkinson’s Disease

**DOI:** 10.3390/s150923727

**Published:** 2015-09-17

**Authors:** Mevludin Memedi, Aleksander Sadikov, Vida Groznik, Jure Žabkar, Martin Možina, Filip Bergquist, Anders Johansson, Dietrich Haubenberger, Dag Nyholm

**Affiliations:** 1 School of Technology and Business Studies, Computer Engineering, Dalarna University, Falun SE-791-88, Sweden; 2 Artificial Intelligence Laboratory, Faculty of Computer and Information Science, University of Ljubljana, Ljubljana 1000, Slovenia; E-Mails: aleksander.sadikov@fri.uni-lj.si (A.S.); vida.groznik@fri.uni-lj.si (V.G.); jure.zabkar@fri.uni-lj.si (J.Ž.); martin.mozina@fri.uni-lj.si (M.M.); 3 Department of Pharmacology, Sahlgrenska Academy, University of Gothenburg, Gothenburg 405 30, Sweden; E-Mail: filip.bergquist@pharm.gu.se; 4 Department of Clinical Neuroscience, Neurology, Karolinska Institutet, Stockholm 171 76, Sweden; E-Mail: anders.johansson@ki.se; 5 Clinical Trials Unit, Office of the Clinical Director, NINDS Intramural Research Program, National Institutes of Health, 10 Center Drive, Rm 6C-5700, Bethesda, MD 20892, USA; E-Mail: haubenbergerd@ninds.nih.gov; 6 Department of Neuroscience, Neurology, Uppsala University, Uppsala 751 85, Sweden; E-Mail: dag.nyholm@neuro.uu.se

**Keywords:** bradykinesia, digital spiral analysis, dyskinesia, machine learning, motor fluctuations, objective measures, Parkinson’s disease, remote monitoring, time series analysis, visualization

## Abstract

A challenge for the clinical management of advanced Parkinson’s disease (PD) patients is the emergence of fluctuations in motor performance, which represents a significant source of disability during activities of daily living of the patients. There is a lack of objective measurement of treatment effects for in-clinic and at-home use that can provide an overview of the treatment response. The objective of this paper was to develop a method for objective quantification of advanced PD motor symptoms related to off episodes and peak dose dyskinesia, using spiral data gathered by a touch screen telemetry device. More specifically, the aim was to objectively characterize motor symptoms (bradykinesia and dyskinesia), to help in automating the process of visual interpretation of movement anomalies in spirals as rated by movement disorder specialists. Digitized upper limb movement data of 65 advanced PD patients and 10 healthy (HE) subjects were recorded as they performed spiral drawing tasks on a touch screen device in their home environment settings. Several spatiotemporal features were extracted from the time series and used as inputs to machine learning methods. The methods were validated against ratings on animated spirals scored by four movement disorder specialists who visually assessed a set of kinematic features and the motor symptom. The ability of the method to discriminate between PD patients and HE subjects and the test-retest reliability of the computed scores were also evaluated. Computed scores correlated well with mean visual ratings of individual kinematic features. The best performing classifier (Multilayer Perceptron) classified the motor symptom (bradykinesia or dyskinesia) with an accuracy of 84% and area under the receiver operating characteristics curve of 0.86 in relation to visual classifications of the raters. In addition, the method provided high discriminating power when distinguishing between PD patients and HE subjects as well as had good test-retest reliability. This study demonstrated the potential of using digital spiral analysis for objective quantification of PD-specific and/or treatment-induced motor symptoms.

## 1. Introduction

Parkinson’s disease (PD) is a neurodegenerative disorder characterized by the gradual onset of muscle rigidity, bradykinesia, *i.e.*, slowness of movement, and, in some patients, tremor. In addition, non-motor symptoms are common. The symptoms may be alleviated by treatments aiming at stimulating dopamine receptors in the brain. The dopamine precursor levodopa is the most effective drug [1]. Advanced therapies aimed at smoothing out dopaminergic stimulation, such as levodopa-carbidopa intestinal gel infusion (LCIG), continuous subcutaneous apomorphine infusion or deep brain stimulation can be highly successful in treating motor fluctuations. In early stages of PD, it can normalize motor function almost completely, but the duration of action gets shorter as the disease progresses. In the advanced stage of PD, fluctuations in motor performance throughout the day can be a major problem. Previously adequate doses of levodopa are no longer enough to treat the Parkinsonian symptoms, and the patient experiences the “Off” state. Higher doses bring the patient to the desired “On” state but contribute to the development of involuntary movements, *i.e.*, dyskinesia, mainly of the choreatic type. Therefore, optimization of therapy in terms of dosage and dosing frequency is a challenge when the patient has reached the state of motor fluctuations.

The physician’s decision to adjust medication is largely based on historical information from the patient, regarding motor function during activities of daily living. This information may be affected by recall bias, present motor and non-motor symptoms, and non-recognition of certain motor states [2]. Home diaries, on paper, are frequently used in clinical studies but lack reliability because data entries are not time stamped. This may be overcome by using electronic diaries [3]. In clinical practice, in-clinic observation is sometimes used by using clinical rating scales such as the Unified PD Rating Scale (UPDRS) [4] and Hoehn and Yahr staging scale [5]. However, such in-clinic observations are costly, time-consuming, may give misleading findings because the hospital environment is different from the ordinary life environment, and some of the items of the scales have low inter-clinician agreements. Subjective information from the patient should ideally be complemented by objective motor tests. Introduction of reliable and sensitive objective measures of symptom severity and treatment-induced motor symptoms has the potential to aid the diagnosis as well as enhance the clinical management of PD patients. Separating dyskinesia (associated with the over-medicated motor state) from bradykinesia (associated with the under-medicated motor state) using an objective assessment method without the need for a scoring physician would allow for prospective, continuous documentation of motor fluctuations, and therefore be central for assessment and adjustment of therapy. Methods for objective assessment of PD motor symptoms have been successfully validated against conventional PD rating scales [6,7,8,9,10].

As reviewed elsewhere [6], objective assessments of motor symptoms of PD patients from their home environments have been previously tested using different sensor technologies mainly employing wearable sensors. From a clinical perspective, the main focus of interest was on quantifying the severity of specific symptoms like freezing of gait [11,12], dysarthria [13], tremor [14,15,16], bradykinesia [17,18,19] and dyskinesia [18,20,21]. As an alternative to these sensor-based systems, some research groups have focused on quantitative assessment of PD motor symptoms based on touch screen devices [22,23,24]. The touch screen devices record movements caused by the pen tip with great spatial and temporal precision and allow for quantitative characterization of kinematics of upper limb motor performance.

The analysis of spirals, drawn either on paper or on a graphics tablet, has been shown to be a sensitive and valid method to quantify tremor in cohorts of patients with Essential Tremor (ET), which is largely based on the assessment of rhythmical components of tremor within spirals [25,26]. Furthermore, non-tremor spiral abnormalities including smoothness and drawing speed were proposed to be a useful measure that can be derived from spirals in patients with PD as well as other movement disorders [22]. Digital spiral analysis is a promising and inexpensive technique that can be used to quantify movement abnormalities in functionally relevant tasks involving penmanship. Movement scores obtained from spirals have been shown to be correlated well with entire rating scales in ET, and therefore could be seen as neurophysiological surrogate markers for movement abnormality [26]. Previous studies have evaluated the use of digital spiral analysis for quantifying upper limb motor performance of PD patients [22,23,24], but there has been less focus on simultaneous quantification of PD-specific and/or treatment-induced motor symptoms. To our knowledge, only one study [24] addressed the issue of clinical relevance of digital spiral analysis for quantifying the severity of drug-induced dyskinesia by correlating quantitative measures of spirals to UPDRS and a scale that specifically measured dyskinesia. However, in the study performed by Liu *et al.* [24], the focus was only on quantification of dyskinesia represented by a single measure based on standard deviation of frequency transformed drawing velocity signals, without any reference to PD-specific symptoms such as bradykinesia.

In this paper, we propose a data-driven method for objective recognition and classification of PD motor symptoms related to both Off episodes (bradykinesia) and peak-dose dyskinesia. The method is based on several spatiotemporal features calculated from digitized upper limb movement data collected by a touch screen telemetry device. The aims of our study were to: (1) validate the method against ratings on animated spirals scored by four movement disorder specialists; (2) assess the ability of the method to discriminate between PD patients and healthy elderly subjects; and (3) assess test-retest reliability of the method.

## 2. Materials and Methods

### 2.1. Subjects

A retrospective analysis was conducted on recordings from 65 patients with advanced idiopathic PD from nine different clinics in Sweden, recruited from January 2006 until August 2010 [27]. The patients were either treated with LCIG or were candidates for receiving this treatment, which specifically targets motor fluctuations. Written informed consent was given and the regional ethical review board (in Stockholm, Sweden), after reviewing the study protocol, determined that their approval was not required. In addition to the patient group, 10 HE subjects were recruited as baseline comparators. Table 1 presents characteristics of patients and HE subjects.

**Table 1:** Characteristics of Parkinson’s disease (PD) patients and healthy elderly (HE) subjects, presented as median ± inter-quartile range.

	PD Patients	HE Subjects
Patients (n, gender)	65 (43 m, 22 f)	10 (5 m; 5 f)
Age (years)	65 ± 11	61 ± 7
Years with levodopa	13 ± 7	NA
Hoehn and Yahr stage at present	2.5 ± 1 *	NA
Total UPDRS	49 ± 20.5 *	NA

* Clinical assessments performed in the afternoon at the start of each test period. Abbreviation: UPDRS, Unified PD Rating Scale; NA, not applicable.

### 2.2. Experimental Setup

**Figure 1:**
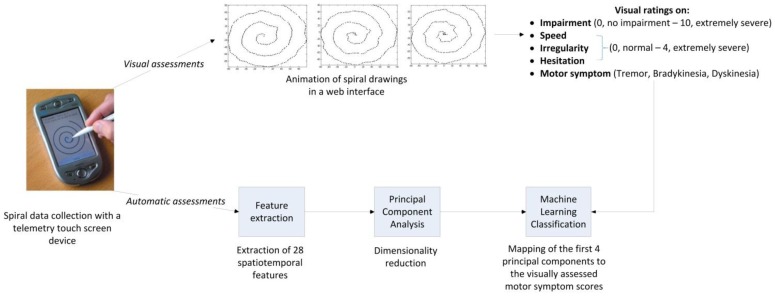
Schematic of the experimental setup.

The experimental study was conducted in the following steps (Figure 1). The spiral data was collected in home environment settings of patients by means of a touch screen telemetry device. The patients performed the tests without direct clinical supervision by the health care providers and, for this reason, there were no simultaneous clinical annotations whether the patients were in Off or dyskinesia motor state at the times they performed the tests in their home environments. To clinically annotate the motor symptoms of the patients, a data visualization approach was employed where a selected sample of spirals was displayed on a web interface. Movement disorder specialists used the web interface and could observe animated versions of the spirals and rate a number of items related to the motor function of the patients. To automate the process of the scoring of the symptoms, a data-driven method was developed by first extracting several spatiotemporal features from the time series of the spirals and then employing Principal Component Analysis (PCA) and machine learning classifiers. Each of the steps is explained in details as below.

### 2.3. Digital Spiral Data Collection

Upper limb movement data were collected using a touch screen telemetry device from home environments of the subjects [28]. Patients used the device quarterly for the first year and biannually for the second and third years. Measurements with the device were performed four times per day during week-long test periods. On each test occasion, the subjects were asked to trace pre-drawn Archimedes spirals, using the dominant hand. The pre-drawn spiral was shown on the screen of the device. The instructions to the subjects were to trace the spiral, using an ergonomic pen stylus, from the center and out, as accurately and fast as possible, supporting neither hand nor arm, with the device placed on a table and to be seated in a chair. The spiral test was repeated three times per test occasion and they were instructed to complete it within 10 s. The device had a sampling rate of 10 Hz and measured both position and time-stamps (in milliseconds) of the pen tip.

### 2.4. Visual/Clinical Assessments of Motor Symptoms

**Figure 2:**
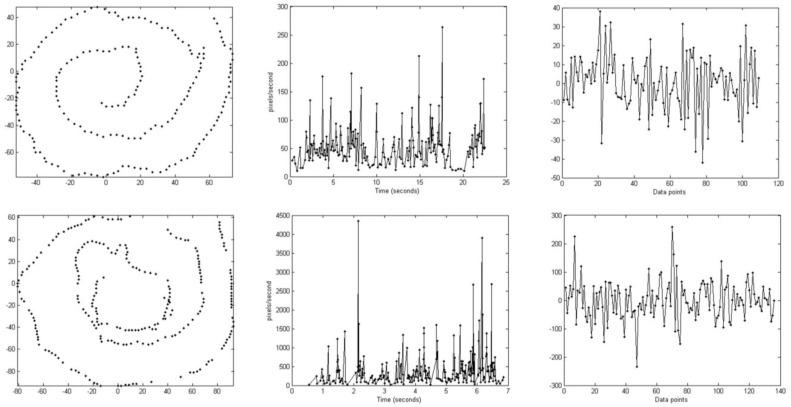
Two illustrative examples of spirals rated as bradykinesia (**upper row**) and dyskinesia (**lower row**) by the four raters. The first column shows the actual spiral drawings, the second shows drawing speed over the test trial and the third column shows the high-frequency wavelet coefficients of radial velocity within the frequency range 2.5–5 Hz. Note the different Y axis scales for the two cases. Mean visual ratings of the four raters for the two spirals were as follows: “impairment” (bradykinesia = 4.75, dyskinesia = 7), “speed” (2.25, 0), “irregularity” (2, 2.75), and “hesitation” (1.5, 0.25). The spirals had the following feature values: mean drawing speed (bradykinesia = 55.8, dyskinesia = 289), standard deviation of wavelet coefficients (14.8, 69.3), Approximate Entropy of drawing speed (0.06, 0.52), and total symmetry (0.18, 0.04).

Four independent raters (Rater1, Rater2, Rater3 and Rater4) used a web interface that animated the spiral drawings and allowed them to observe different kinematic features during the drawing process and to rate task performance [29]. Initially, a number of kinematic features were assessed including “impairment”, “speed”, “irregularity” and “hesitation” followed by marking the motor symptom on a 3-category scale: tremor, bradykinesia and/or choreatic dyskinesia. The kinematic features were considered specific for the type of upper limb motor movements found in PD patients suffering from motor fluctuations. “Impairment” was rated on a scale from 0 (no impairment) to 10 (extremely severe) whereas “speed”, “irregularity” and “hesitation” were rated on a scale from 0 (normal) to 4 (extremely severe). “Impairment” is the overall impression of the shape of the spiral in relation to the pre-drawn sample. “Speed” refers to the impression of average speed of moving the pen tip over the touch screen surface. Unusually, fast drawing was considered normal (0) and scale steps 1–4 were used to describe slowness of movement. “Irregularity” refers to the impression of spatial deviation from the projected drawing movement. “Hesitation” could either be impression of start hesitation, *i.e.*, difficulty of initiating the movement, or arrests during drawing. The motor symptom is the clinical interpretation of the main reason for any abnormality in these categories, taken together. Figure 2 shows two spirals rated as bradykinetic and dyskinetic, as rated by the four raters.

The web interface was designed as a three-tier web application using JavaServer Pages as a code-behind model and MySQL as a back-end database. The application initially retrieved *x*, *y* and *timestamp* of the spiral from the database tables and then animated the drawing in real-time using the Canvas element of HTML5. The visual rating process was divided into two phases: the training phase and rating phase. Initially, one of the raters browsed through the database and rated a sample of spirals being either bradykinetic or dyskinetic. The examples were chosen from test occasions where similar motor symptom was determined for all the three spirals. The rationale for focusing only on these two motor symptom categories was that the patients included in this study did not have action tremor. In the training phase, the other three raters observed these pre-selected cases and their animations. In the subsequent rating phase, all four raters assessed the spirals shown to them and the ratings were recorded. All four raters were shown the same collection of cases; each case consisting of three spiral drawings. This collection was a random subset of all spiral recordings in the database, where the three spiral drawings from three selected test occasions per patient were animated. The ratings recorded in the rating phase served to analyze the inter-rater agreements and as inputs for the machine learning experiments.

### 2.5. Automatic Scoring of Motor Symptoms

#### 2.5.1. Feature Extraction

The features are based on different kinematic quantities of spirals including radius, angle, speed and velocity with the aim of measuring the severity of involuntary symptoms and discriminate between PD-specific (bradykinesia) and/or treatment-induced symptoms (dyskinesia). The following features were calculated and used in subsequent analysis.

The first three features relate to first three statistical moments of a time series signal. The mean drawing speed (Sp.mean) is calculated to represent the first raw moment and is defined as the mean rate of change of position with time, using the following equation:

(1) $$Sp.mean=\;\overset{n}{\underset{i=1}{\sum }}\frac{\sqrt{{\left(x_{i+1}-x_{i}\right)}^{2}+{\left(y_{i+1}-y_{i}\right)}^{2}}}{t_{i+1}-t_{i}}$$

where ***n*** is the total number of spiral data points, ***x*** is the horizontal coordinate of pixels on the screen, ***y*** is the vertical coordinate and ***t*** is the time in seconds. The second and third moments include coefficient of variation of speed (Sp.cv, defined as the ratio between standard deviation and mean) and skewness of speed (Sp.skew), defined as:

(2) $$Sp.skew=\;\frac{\left(\frac{\sum_{i=1}^{n}{\left(x_{i}-\bar{x}\right)}^{3}}{n}\right)}{s^{3}}$$

where ***x*** is the actual data point in the time series, $\bar{x}$ is the mean, ***s*** is the standard deviation and ***n*** is the total number of data points. In order to quantify the amount of hesitation or absence of movement during the spiral drawing process, mean delta time (Dt.mean) is calculated as the change in time defined as $t_{i+1}-t_{i}$. Then, a feature analogous to the component of drawing velocity away from or toward the spiral origin is calculated by taking the coefficient of variation of high-frequency components from 2.5 to 5 Hz (According to Nyquist criterion, 5 Hz is the maximum frequency component that can be detected in our signals). Initially, radial velocity (***RV***) is calculated, using the following equation:

(3) $$RV=\;\frac{r_{i+1}-r_{i}}{t_{i+1}-t_{i}}$$

where ***r*** is the radius defined as $\sqrt{\left(x^{2}+y^{2}\right)}$, and then used in a 1-level Discrete Wavelet Transform (DWT) using Daubechies 10 wavelet function. The coefficient of variation of the high-frequency wavelet coefficients (Rv.hf.cv) was finally calculated and used as a feature in subsequent analysis for detecting the variation in short and fast movement changes during the spiral drawing task. In order to capture global minima and maxima in drawing speed, minimum and maximum values were obtained from the high-frequency wavelet coefficients after applying 1-level DWT on the drawing speed signal. Hence two more features are defined and denoted as (Ds.hf.min) and (Ds.hf.max), respectively. To quantitatively measure changes in sequential irregularity during the drawing process, Approximate Entropy (ApEn) was applied on the radial velocity and drawing speed. The ApEn is a non-linear measure which quantifies the similarity between a chosen window of time series of a given duration and the next set of windows of the same duration [30]. A time series containing a single frequency component has a relatively small ApEn value, whereas more complex time series containing multiple frequency components are associated with high ApEn values, indicating a high level of irregularity. The ApEn requires specification of two parameters including the length of the window ***m*** and a measure of similarity ***r***, each of which must remain fixed during all calculations. In our case, ***m*** was set to 2 and ***r*** to 0.2 (*i.e.*, 20% of the standard deviation of the time series), as suggested by Pincus [28]. Hence, two more features denoted as Rv.apen and Ds.apen were calculated and used subsequently.

A superset of the following features was previously developed and used for detecting signs of PD and ET tremors [31]. Features used for the analysis in this paper are described here for reader’s convenience while a more detailed description of them can be found elsewhere [32]. Initially, angular velocity (*AV*) was calculated and then analyzed for local extremes (minima and maxima). The *AV* was calculated using the following equation:

(4) $$AV=\frac{\varphi_{i+1}-\varphi_{i}}{t_{i+1}-t_{i}}$$

where **φ** is the angle represented by a four-quadrant inverse tangent of ***X*** and ***Y*** coordinates after the phase angle was corrected. The extremes in the time series signals represent the changes in direction (increase/decrease). For instance, when using the absolute drawing speed, the extremes represent the time points when the subject started to accelerate or decelerate, which can be useful for quantifying arrests during drawing. The extremes were extracted along with the first and the last points of the time series. The absolute differences between each two neighboring extremes were then calculated and referred to as **Δ*Peaks***. The feature ***nPeaks*** is the number of extremes normalized with the length of the time series signal. The mean and standard deviation of **Δ*Peaks*** were calculated and denoted as ***avgP*** and ***stdP***, respectively. The change of variation over time in **Δ*Peaks*** during test trial was calculated using a sliding window, which compared standard deviations over 30 overlapping windows. The new feature denoted as ***incDev*** is based on the Spearman’s rank correlation coefficient of the level of variation with time. The rationale for including the ***incDev*** feature is based on the hypothesis that the deviations could be more pronounced on the more convoluted parts of the spiral (it is easier to make a significant error on the smaller, inner parts of the spiral). Finally, ***avgP***, ***stdP*** and ***incDev*** were applied to absolute drawing speed, ***RV***, ***AV***, radius and angle over time. More specifically, the following features were calculated and used in subsequent analysis. Radius features included: avgP of radius (R.avgP), incDev of radius (R.incDev), nPeaks of radius (R.nPeaks) and stdP of radius (R.stdP). Absolute drawing speed features included: avgP of absolute drawing speed (Ads.avgP), nPeaks of absolute drawing speed (Ads.nPeaks) and stdP of absolute drawing speed (Ads.stdP). Radial velocity, ***RV*** features included: avgP of ***RV*** (Rv.avgP), nPeaks of ***RV*** (Rv.nPeaks), stdP of ***RV*** (Rv.stdP) and a specific feature denoted as Rv.pn005. The Rv.pn005 feature measures the percentage of the spiral length when the subject drew towards the center of the spiral. More specifically, the feature represents the percentage of the length when ***RV*** is below −0.05, *i.e.*, slightly lower than zero for accounting potential noise/measurement error. When drawing an ideal Archimedean spiral, the radius constantly increases meaning that the drawing is never directed towards the center. Obviously, even the healthy subjects may occasionally have to correct the direction and draw towards the center. However, this movement deficit is much more prominent in PD patients who have impaired upper limb motor movements. Angular velocity, ***AV*** features included: avgP of ***AV*** (Av.avgP), nPeaks of ***AV*** (Av.nPeaks) and stdP of ***AV*** (Av.stdP). The incDev of angle over time (Angle.incDev) was calculated and was used in subsequent analysis.

Another feature measures the asymmetry of the spirals. Initially, the asymmetries in horizontal (*X*) and vertical (*Y*) directions were calculated followed by calculating the total asymmetry after taking the sum in both directions. For instance, given the origin of the spiral, the horizontal asymmetry was calculated as the difference between the distance of the leftmost point of the spiral from the vertical axis and the distance of the rightmost point of the spiral from the vertical axis, divided by the sum of the two distances. Division by the sum of the distances was performed in order to normalize the asymmetry feature with the overall size of the spiral in the given direction. The vertical asymmetry is calculated analogously. The total asymmetry (TOTSYMM) was defined as the sum of the asymmetries in both directions.

The following three features measure the “error” the subject makes while drawing the spiral; the error is perceived in terms of spatial deviations from the ideal spiral. These features measure the error in polar coordinate space. Least squares fit of the drawn spiral in polar coordinates gives us the optimally fitting ideal spiral. A new feature called Err was defined as the root mean squared error (RMSE) between the ideal and drawn spirals. In a similar way, the feature, denoted as Err0, was constructed. The only difference is that we set a constraint to the fit line to pass through the coordinate system origin (the origin is set to the first point of the drawn spiral). However, the spiral may be perfect, but users often have problems with the start of the drawing. This in turn results in the spiral that is translated from the optimal origin. To measure the deviation due to translation, we compute the virtual optimal origin of the user’s spiral, Ou, translate it to (0, 0). Ou is the origin of the spiral that is virtual best fit to the user’s spiral. We use differential evolution algorithm [33] to determine Ou. The feature errBF is RMSE between the ideal and the translated drawn spiral. The feature err0BF is the same, except that the ideal spiral has to pass through (0, 0).

#### 2.5.2. Principal Component Analysis

In the current work, the PCA, using the correlation matrix method, was applied on the 28 features. The most commonly used criterion for retention of the principal components (PCs) is to select the PCs that account for more than 70% of the total variance. In our case, we selected the first four PCs and the cumulative proportion of variance explained by them was 69%. More PCs were not selected since a simpler model was favored. The aim of the PCA was to remove the multi-collinearity among the 28 features and thus improve the performance of the machine learning classifiers.

#### 2.5.3. Classification

For motor symptom (bradykinesia *vs.* dyskinesia) classification, a stratified 10-fold cross validation technique was employed. We experimented with five machine learning methods from the Weka data-mining toolkit [34]: Multilayer Perceptron (MLP), linear and non-linear Support Vector Machines (SVM), Random Forests (RF) and Logistic Regression (LR). All methods were trained with default parameters, except for RFs, where the number of trees was increased to 100. The learned classifiers were used to classify the presence/absence of slowness of movements and abrupt, involuntary movements, respectively. The first four PCs were used as inputs to the classifiers and were to be mapped to the corresponding visually assessed motor symptom scores (bradykinesia or dyskinesia). The inputs to machine learning classifiers were selected as follows: each learning/testing example consisted of the four PCs and the rating of motor symptoms (bradykinesia or dyskinesia) acting as the class. As each case was assessed by several raters, only one of the ratings was chosen—this was selected at random. This randomization ensured that all four raters were equally represented in the learning/testing data. Only cases (109 out of 358) that had a mean impairment category of greater than or equal to 5 (on a scale from 0, no impairment to 10, extremely severe), as assessed by a computer method [23], were included in the classification analysis. The rationale for including only spirals with moderate to severe impairments was that in these spirals it was easier to identify spatiotemporal motor deficits that were specific to bradykinesia and dyskinesia. This applied during both visual and computer classifications.

#### 2.5.4. Statistical Analysis

Agreements between the four raters on visual scores were assessed by intra-class correlation (ICCs) coefficients after taking the mean of all six possible correlations and Weighted Kappa statistics. Correlations between the individual first four PCs and mean visual ratings of the individual kinematic features were assessed by Spearman rank correlations. Agreements between the machine learning classifiers and the four raters when assessing the motor symptoms (bradykinesia *vs.* dyskinesia) were assessed by a number of metrics including classification accuracy (%), sensitivity, specificity, Weighted Kappa and area under the receiver operating characteristics curve (AUC). The test-retest reliability of the four PCs was assessed after calculating correlations between the PCs for the three spiral test trials and then calculating the mean of all the possible correlations. The linear-mixed effects (LME) models were used to assess the discriminative ability of the four PCs when differentiating patients from HE subjects. The LME models were based on a restricted maximum likelihood estimation method with subject ID as a random effect and group as a fixed effect of interest. The within-subject variability of the four PCs was assessed by ICCs derived from the LME models, for the two subject groups.

## 3. Results

### 3.1. Inter-Rater Agreements

Agreements between the four raters when rating the individual kinematic features were as follows: ICC of 0.78 (range, 0.67–0.92) for “impairment”, 0.74 (0.68–0.81) for “speed”, 0.70 (0.66–0.74) for “irregularity”, and 0.49 (0.4–0.64) for “hesitation”. The raters could identify the motor symptom in 313 cases out of total 358 (87%). Out of these rated test occasions, 16 test occasions were rated with motor symptom as tremor whereas the rest were either rated as bradykinesia or dyskinesia. There were only two test occasions for which all the four raters either classified them as tremor or could not identify the motor symptom. Tremor spirals were rhythmical and were associated with some amount of hesitation. The spirals with non-identifiable motor symptom had similar spatiotemporal characteristics as the spirals drawn by HE subjects with minor irregularities and normal speed. When assessing the two main motor symptom categories (bradykinesia or dyskinesia) in animated spirals, the agreements between the four raters ranged from fair to substantial (Table 2).

**Table 2:** Agreements (Weighted Kappa statistics; percentage agreement; false positive rate; false negative rate) between the four raters when rating the motor symptom (bradykinesia and dyskinesia) in animated spirals. All Kappa statistics are highly significant (each *p* < 0.001).

	Rater 1	Rater 2	Rater 3
Rater 2	0.52; 76.1; 25; 22.5		
Rater 3	0.43; 71.8; 1.9; 56	0.48; 76.7; 3.3; 51	
Rater 4	0.23; 62.4; 42.3; 0	0.26; 66.4; 42.3; 0	0.63; 89.3; 12.1; 0

### 3.2. Correlations/Agreements between Computed and Visual/Clinical Scores

There were good correlations between mean ratings of the four raters on individual kinematic features and computed scores (Table 3). The PC1 correlated significantly with the mean visual ratings of all the kinematic features, except “hesitation”. Nevertheless, mean visual ratings of “hesitation” were significantly correlated with the remaining PCs *i.e.*, PC2, PC3 and PC4. Among the four visually rated kinematic features, “speed” was the most correlated to all four PCs.

**Table 3:** Absolute Spearman rank correlations between the first four principal components (PCs) and mean visual ratings of individual kinematic features.

	PC1	PC2	PC3	PC4
Impairment	0.56	0.03	0.1	0.17
Speed	0.58	0.53	0.51	0.43
Irregularity	0.69	0.24	0.03	0.03
Hesitation	0.08	0.34	0.29	0.33

Table 4 shows the evaluation of several classification algorithms on our data set. The MLP and the RF classifiers obtained comparable results, while the results of linear SVM and LR were a bit worse. The best performing classier (MLP) classified the motor symptom that is bradykinesia or dyskinesia with an accuracy of 84%.

**Table 4:** Classification accuracies (%), Weighted Kappas and area under the receiver operating characteristics curve (AUCs) of different classifiers trained to distinguish between bradykinesia and dyskinesia. All scores were estimated with a stratified 10-fold cross-validation.

	MLP	RF	SVM (Radial Basis Function Kernel)	SVM (Linear)	LR
Accuracy	84	83	79	76	76
Weighted Kappa	0.65	0.60	0.50	0.47	0.47
AUC	0.86	0.85	0.74	0.74	0.83

Table 5 presents the results of classification of motor symptoms with the MLP, including the accuracy, sensitivity, specificity, Weighted Kappa and AUC.

**Table 5:** Assessments of motor symptoms for the multilayer perceptron (MLP) classifier and the four raters. The sample used for this analysis consisted of randomly selected cases that were rated from the four raters. The computed scores are derived after applying stratified 10-fold cross validation on the MLP classifier. Sensitivity shows the performance of Bradykinesia class. Specificity shows the performance of Dyskinesia class. Abbreviation: AUC, area under the receiver operating characteristics curve; CI, confidence interval.

	MLP Classifier			
		Bradykinesia	Dyskinesia	Total
Raters	Bradykinesia	28	8	36
	Dyskinesia	9	64	73
	Total	37	72	109
Accuracy	84%			
Sensitivity	75.7% (CI: 58.8%–88.2%)			
Specificity	88.9% (CI: 79.3%–95.1%)			
Weighted Kappa/AUC	0.65/0.86			

### 3.3. Test-Retest Reliability of the Computed Scores

The test-retest reliability of the four PCs across the three spiral test trials was as follows: 0.75 for PC1, 0.6 for PC2, 0.64 for PC3, and 0.39 for PC4. These results indicate that the derived PCs are stable and consistent over time.

### 3.4. Separation of Healthy Elderly Subjects from PD Patients

**Figure 3:**
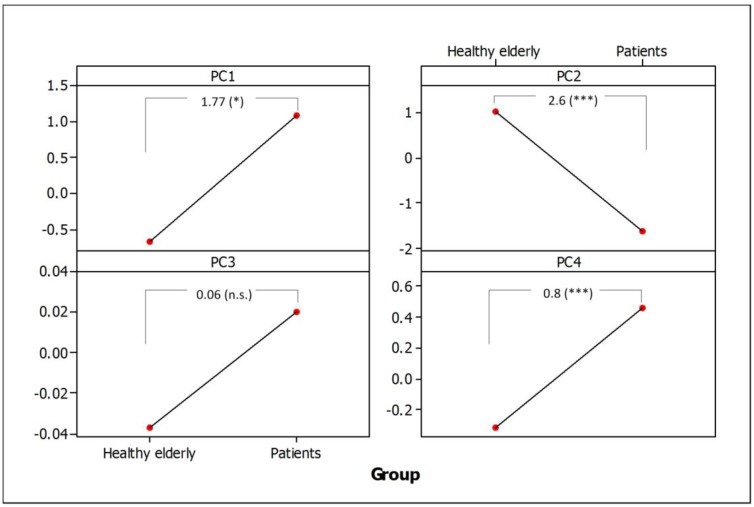
Mean scores of the first four PCs for healthy elderly subjects and PD patients, corrected for within-individual variation using the LME models. Absolute mean differences and P-values are shown with respect to the healthy elderly group. Symbols: * = *p* < 0.05, *** = *p* < 0.001, n.s. = not significant. Group: healthy elderly (*n* = 876), patients (*n* = 545).

Figure 3 shows the mean scores of the four PCs for patients and HE subjects. Significant differences were found between the two groups in all the PCs, except for the PC3. The PD patients also had higher within-subject variability in their PCs compared to HE subjects with ICCs (patients *vs.* HE subjects) of (0.51 *vs.* 0.67) for PC1, (0.62 *vs.* 0.85) for PC2, (0.56 *vs.* 0.71) for PC3 and (0.69 *vs.* 0.88) for PC4, where a high ICC value indicates low within-subject variability.

## 4. Discussion and Conclusions

In this paper, we propose a method for quantifying PD motor symptoms related to Off episodes and peak dose dyskinesia, which are prominent in advanced patients experiencing motor fluctuations. The symptoms are quantified by calculating several spatiotemporal features from spiral recordings gathered by a telemetry touch screen device. Based on the features, several classifiers were employed to characterize the severity of the motor symptoms and classify them into PD-specific and or/treatment-induced in relation to visual assessments of four movement disorder specialists. The best performing method was the MLP classifier, which classified spirals as being either bradykinetic or dyskinetic with an accuracy of 84% and sensitivity and specificity of 75.7% and 88.9%, respectively. In addition, the method had good test-retest reliability and provided high discriminating power when distinguishing between movement patterns exhibited by PD patients, irrespective whether their motor symptom was rated as bradykinesia or dyskinesia, and HE subjects.

During clinical/visual assessments of animated spirals, variations in drawing speed were the core features when separating bradykinesia from dyskinesia. In dyskinesia cases, speed increased without previous slowing/arrest whereas in bradykinesia cases speed increased with slowing/arrest before onset as a result of festination of drawing. The speed features emerged as the most important features for classification of motor symptoms since the four PCs were significantly correlated to the mean rating of the “speed” kinematic feature (Table 3). Spirals rated as dyskinesia on average were more impaired than spirals rated as bradykinesia. When comparing mean scores of drawing impairment in spirals, as calculated by a computer method [23], in relation to visual ratings of the two motor symptoms for the four raters the spirals rated as dyskinetic were more impaired than those rated as bradykinetic. The rationale for including only spirals with moderate to severe impairment in the machine learning classification was based on the fact that only on those cases movement patterns associated to dyskinesia and/or dyskinesia could be visually detected. Moreover, the four movement disorder specialists had better agreements when they rated cases with moderate to severe impairments as compared to when they rated cases with mild impairment. Spiral drawing performance was also shown to be more impaired when patients self-assessed their motor state as dyskinetic than when they assessed their state as Off [35].

A limitation of the present study is that the proposed method is not designed for detecting and quantifying tremor This is because the advanced patients in the Swedish study did not usually have action tremor which was expected due to a lower prevalence of action tremor in PD as compared to bradykinesia and dyskinesia. The raters identified spirals as tremor in 16 out of a total of 313 cases. The spatial and temporal characteristics of these cases were different from the cases rated as bradykinesia and dyskinesia. In contrast to bradykinesia and dyskinesia spirals, tremor spirals were associated with rhythmic oscillations. From these 16 tremor cases, tremor components were prominent only when movements slowed down, a phenomenon that could be related to postural tremor in this patient group. This limitation is also related to the low sampling frequency of the telemetry device. Action tremor in PD is usually in the 6–10 Hz range [24] and with a sampling rate of 10 Hz it is not possible to clearly detect tremor amplitudes. In the future, it would be interesting to collect digital spiral data from PD patients with action tremor, using a device with a higher sampling rate. This would allow us to investigate the feasibility of more spatiotemporal features designed for capturing the whole spectrum of PD motor symptoms including both bradykinesia and tremor as well as dyskinesia. In contrast to the methods presented in [19,20,21], and [24], which measure only one of the motor symptoms that is either bradykinesia or dyskinesia, our method measures both of the symptoms. This was achieved by first calculating features that specifically measured the symptoms and then combining them during the dimension reduction procedure and machine learning classification. The method developed by Griffiths *et al.* [18] was based on wearable sensors and could model both bradykinesia and dyskinesia. In contrast to their method, which was an expert system based on fuzzy logic, our method combines several spatiotemporal features and weighs them by data-driven machine learning methods. The strength of our system is the ability to document motor symptoms at certain time intervals in a longitudinal manner. Repeatedly drawing spirals on a touch screen device is more convenient than using paper home diaries, provides reliable data for analysis and also provides a higher time resolution.

Another limitation of the study is that the clinical interpretation of the motor symptoms of the patients was done by visual inspection of their animated spirals and not by live or video clinical observations. Since patients repeatedly used the device at their homes without clinical supervision, the rationale for visualizing spiral performance was to derive a target measure, which can be used during training and testing of the machine learning classifiers. Given the fact that the clinical ratings based on UPDRS and Hoehn and Yahr staging scale were performed only on test period level and there is large within-patient variability in motor fluctuations in this patient group, they could not be used for assessing the validity of the method. Nevertheless, from Table 2, it can be noted that inter-rater agreements when visually rating the two main categories of the motor symptoms were fair to substantial, having Weighted Kappa coefficients on a range from 0.23 to 0.63. The inter-rater variabilities from the mean rating could be reduced by including more raters. In our comparison of rating motor symptoms by the raters and by the machine learning classifiers, we have used a randomized sample from the four raters. Assuming that the approach to visual rating of motor symptoms from animated spirals is novel and not yet validated, we randomly pooled cases from the four raters and presented them to the classifiers with a stratified 10-fold cross-validation for automating the process of scoring bradykinesia and dyskinesia. In this way, we ensure a proper representation of cases from the four raters in both training and testing sets. The Weighted Kappa coefficient between the ratings of the four raters and the MLP classifier was 0.65; slightly higher than the maximum coefficient found among the four raters. These results indicate that the method can reasonably well replicate visual interpretations of spirals by movement disorder specialists. In addition, particular strengths of the method include separation between voluntary movements exhibited by HE subjects and slow/involuntary movements found in PD patients (Figure 3), and detection of differences in variabilities during spiral drawing between HE subjects and patients. The rationale behind including the HE subjects dataset in the analysis was to assess the ability of the method to distinguish between movement exhibited by PD patients and healthy subjects during spiral drawing tasks. Since the number of subjects in the two groups was not equal, we employed LME models to model within- and between-subject dependencies as well as to account for the unbalanced data design [36]. Usability and compliance of the telemetry device have been assessed previously [37]. The median compliance was 93% indicating that the patients were very compliant with using the telemetry device. User evaluations showed acceptable usability.

Due to the methodological challenge of assessing mild impairment, the next step in our research is to complement digital spiral analysis with more sensor measurements including motion sensors, tapping tests and eye tracking. In addition, we plan to videotape the subjects so that their motor function at the time of drawing can be assessed and blindly rated by independent raters. In this way, we can evaluate the feasibility of animating spirals to clinicians as well as investigate whether digital spiral analysis provides sufficient symptom information when used in combination with other sensors that provide supportive objective data. The plan also includes assessing the sensitivity of the objective measures to detect changes in treatment effects.

In conclusion, the proposed method could reasonably discriminate well between PD-specific and/or treatment-induced motor symptoms, in relation to visual assessments of movement disorder specialists. The objective assessments could provide a time-effect summary score that could be useful for improving decision-making during symptom evaluation of individualized treatment when the goal is to maximize functional On time for patients while minimizing their Off episodes and troublesome dyskinesia.

## Appendix

The animated versions of the two spirals shown in Figure 2 can be accessed online. The spiral rated as bradykinetic can be accessed at [38] whereas the spiral rated as dyskinetic can be accessed at [39].
